# Smokeless tobacco mortality risks: an analysis of two contemporary nationally representative longitudinal mortality studies

**DOI:** 10.1186/s12954-019-0294-6

**Published:** 2019-04-11

**Authors:** Michael T. Fisher, Susan Marie Tan-Torres, Charles L. Gaworski, Ryan A. Black, Mohamadi A. Sarkar

**Affiliations:** 1 Regulatory Affairs, Altria Client Services LLC, 601 East Jackson Street, Richmond, VA 23219 USA; 2 Quality, Altria Client Services LLC, 601 East Jackson Street, Richmond, VA 23219 USA; 3 Kozmin Consulting, LLC, 103 Barclay Ct, Flat Rock, NC 28732 USA

**Keywords:** Smokeless tobacco, Cigarettes, Epidemiology, Mortality hazard ratio

## Abstract

**Background:**

Assessments supporting smokeless tobacco (SLT) disease risk are generally decades old. Newer epidemiological data may more accurately represent the health risks associated with contemporary US-based SLT products, many of which contain lower levels of hazardous and potentially hazardous chemicals compared to previously available SLT products.

**Methods:**

Data from two longitudinal datasets (National Longitudinal Mortality Study—NLMS, and the National Health Interview Survey—NHIS) were analyzed to determine potential associations between SLT use and/or cigarette smoking and all-cause and disease-specific mortality. Mortality hazard ratios (HR) were estimated using a Cox proportional hazards regression model applied to various groups, including never users of any tobacco or SLT product, and current and former SLT users and/or cigarette smokers.

**Results:**

The two datasets yielded consistent findings with similar patterns evident for the specific causes of death measured. All-cause mortality risk for exclusive SLT users was significantly lower than that observed for exclusive cigarette smokers and dual SLT/cigarette users. Similar trends were found for mortality from diseases of the heart, chronic lower respiratory diseases, and malignant neoplasms. Mortality risk for lung cancer in exclusive cigarette smokers was increased by about 12-fold over never-tobacco users but was rarely present in exclusive SLT users in either survey (NHIS, < 5 cases/1,563 observations; NLMS, 3 cases/1,863 observations). While the data in the surveys are limited, SLT use by former cigarette smokers was not associated with an increase in the lung cancer risk HR compared to that by former cigarette smokers who never used SLT.

**Conclusions:**

Emerging epidemiological data provides a new perspective on the health risks of SLT use compared to risks associated with cigarette smoking. HR estimates derived from two current US datasets, which include data on contemporary tobacco products, demonstrate a clear mortality risk differential between modern SLT products and cigarettes. Cigarette smokers had an increased overall mortality risk and risk for several disease-specific causes of death, while SLT users consistently had lower mortality risks.

**Electronic supplementary material:**

The online version of this article (10.1186/s12954-019-0294-6) contains supplementary material, which is available to authorized users.

## Background

There is substantial evidence that tobacco products are hazardous and their use carries the risk of serious disease [1, 2]. However, the type and magnitude of disease risk differs between different tobacco product categories and extent of use [2–5]. For example, researchers in the public health community have qualitatively placed tobacco products on “the continuum of risk,” with conventional cigarettes having the highest risk, an intermediate risk for smokeless tobacco (SLT) products and e-cigarettes, and tobacco use cessation as the lowest risk state [6]. Nutt et al. quantified this risk continuum based on a multi-criteria decision analysis approach, wherein the weighted score for cigarettes was 100 and SLT and other products were scored at less than 15 [7]. However, as described by the authors, a limitation of the study was the “lack of hard evidence for the harms of most products on most of the criteria.”

The purpose of this study is to provide a direct comparison between the health risks of US smokeless tobacco products and conventional cigarettes. Also, this study provides health risk data on tobacco product use states not frequently reported in the published literature. These include health risk estimates for dual users of SLT and cigarettes as well as risk estimates for former cigarette smokers who now use SLT products, which can be considered surrogates for consumers who switch from cigarette smoking to SLT use. These data can be useful in considering the implications of encouraging cigarette smokers who cannot or will not quit using tobacco products to switch to potentially lower risk alternatives such as SLT [8, 9]. For example, tobacco product switching may include a period of concurrent use of both products, such as cigarettes and SLT. From a harm reduction policy perspective, it is important to understand if concurrent use is associated with any additive or synergistic health consequences. Understanding the risks associated with concurrent use of SLT and cigarettes is also important because approximately 30% of SLT users smoke cigarettes, a rate that has been consistent over many years [10]. Also, any risks associated with switching from cigarettes to lower risk alternatives, such as SLT, should be compared with the risks of quitting all tobacco use when considering harm reduction policies. Finally, much of the published epidemiology of US SLT products is somewhat older, and the risks associated with use of tobacco products may change over time as tobacco product designs change [11, 12].

There is a clear need for a more recent evaluation of the health risks associated with SLT use; indeed, a recent publication by Timberlake et al. attempts to address this gap by conducting an analysis of the data from the National Longitudinal Mortality Study (NLMS), one of the same datasets used in the present study, although without the comparison to cigarette smoking we include [13]. In their study, an elevated risk of death due to coronary heart disease among SLT users compared to never-tobacco users was observed but without a significant excess risk for all-cause mortality. The current study adds to the growing body of evidence regarding current US SLT products by providing a comprehensive quantitative comparison of the mortality risks from a second publicly available dataset, the National Health Interview Survey (NHIS), in addition to the NLMS. Together, these surveys contain tobacco use and mortality data, spanning the mid-1980s to 2011, and allow for estimation of mortality risks, which are likely more reflective of the hazards encountered by SLT consumers in the US population using the most currently available products, including moist smokeless tobacco (MST). We use these data to derive mortality risk estimates for specific tobacco use profiles, including exclusive cigarette use, exclusive SLT use, dual cigarette/SLT use, or SLT use by former smokers. We provide evidence on relative mortality hazards regarding exclusive SLT use and cigarette smoking relative to never-tobacco use. Additionally, we assess the risk differential between former smokers currently using SLT relative to former smokers who do not use SLT to address the question of whether beneficial health outcomes related to smoking cessation are maintained if former smokers use SLT.

## Methods

### Source data

We analyzed datasets created by linking nationally representative cross-sectional survey data (i.e., NLMS and NHIS) to National Death Index (NDI) data available from the National Center for Health Statistics (NCHS) [14]. The NDI is maintained by NCHS and contains death certificate information for all decedents in the USA since 1979 [15].

The NLMS is a nationally representative, longitudinal mortality study. The NLMS Public Use Microdata Sample (PUMS) tobacco use (TU) file consists of samples of Current Population Survey Tobacco Use Supplements (CPS-TUS) administered from 1993 through 2005 linked to NDI vital status data. We analyzed version five of the PUMS TU file, which contains demographic, vital status, and tobacco use data for 493,282 CPS-TUS respondents. The PUMS TU data have 5 years of mortality follow-up for all respondents, with each decedent’s underlying cause of death assigned to one of 113 aggregate causes. We limited PUMS TU file analyses to respondents who were at least 18-years old at survey who are never users of either pipe tobacco or cigars, and for whom, analysis weight, follow-up time, vital status, and model covariates are known, yielding 210,090 respondents eligible for our analyses and 8,580 deaths. We excluded pipe and cigar smokers from our analyses because the purpose of this study was to directly compare SLT and cigarettes. Cigars, in particular, are a diverse tobacco product category with highly variable usage patterns, which could render it difficult to properly adjust for this exposure [16]. There were 129 deaths among the 3,492 current SLT users in the analysis dataset. An accounting of the impact of sample limitations is shown in Additional file 1.

The NHIS is an annual, nationally representative survey of the civilian non-institutionalized US population. NHIS surveys from 1986 through 2009 are linked to NDI data by NCHS with vital status follow-up through December 31, 2011. We analyzed both the publicly available data and the restricted data with 6, 10, and up to 24 years of follow-up. We only report data for the 10-year follow-up, since the results did not differ substantially between the various follow-up periods. Access to restricted data is available through application to the NCHS. We included all surveys where smoking, SLT use, pipe use, and cigar use is identified (1987, 1991–1992, 1998, 2000, and 2005); 154,286 people (29,707 deaths) were eligible for restricted access analysis, including 650 deaths among 3,005 current SLT users. An accounting of the impact of sample limitations is shown in Additional file 2.

### Data analysis

We defined nine mutually exclusive tobacco use groups based on self-reported current, former, or never SLT or cigarette use as designated in the NLMS and NHIS data sets. Current smokers were defined as respondents who smoked 100 cigarettes in their lifetime, smoke cigarettes every day or some days (NHIS), or smoked cigarettes now (at the time of the survey) (NLMS). Former smokers were defined as respondents who reported smoking 100 cigarettes but did not smoke cigarettes at the time of the survey [17]. In the NLMS sample, current SLT users were those reporting SLT use every day or some days and former SLT users were those reporting no SLT use at the time of the survey [18]. In the NHIS sample, SLT users were those reporting SLT use (snuff or chewing tobacco) at least 20 times. Among those, current SLT users reported they now used SLT and former users were those reporting they did not currently use SLT.

The surveys did not, in all cases, provide specific information related to the type or extent of SLT use. Questions in the survey vary from year to year and in some years, there are questions asking about age at initiation of SLT use, intensity of use, and duration of use. From the information available related to SLT use, snuff users (*n* = 797) used the product for an average of 22 years at the time of survey (SD = 20 years) while chewing tobacco users (*n* = 1,158) used the product for an average of 25 years (SD = 20 years).

We estimated the mortality hazard ratio (HR) using the Cox Proportional Hazards Model [19], incorporating the following covariates into the model: (1) gender, (2) race, (3) age, (4) BMI—for the NHIS data only (not available in the NLMS dataset), (5) educational attainment, (6) family income, (7) health status, (8) tobacco use, and (9) cigarettes smoked per day. We selected these covariates based on previously published research which used similar models for estimating mortality hazard ratios for other purposes [20–28]. We evaluated the proportionality assumption of the hazard models through tests of the log-log survival curves, scaled Schoenfield residual plots, and standardized score process plots, as a function of follow-up time. The proportionality assumption was generally upheld. The Breslow method of handling ties was used [29]. In the all-mortality model, a person was censored if they are “presumed alive” by the non-match to the National Death Index (when matching variables are present). Data on follow-up mortality is limited to eligible adults who have a valid combination of social security number (SSN), birth data, and name. In the all-cause mortality model, a person is censored if they are “presumed alive” by a non-match to the NDI as determined by the National Center for Health Statistics data linkage team. For the disease-specific mortality model, a person is censored if they are “presumed alive” by the non-match to the National Death Index, as determined by the NCHS data linkage team, or if they die from a cause other than the one being modeled.

We excluded pipe tobacco and cigar users from all analyses. We pooled “snuff” and “chewing tobacco” users in our analysis because snuff and chewing tobacco users tend to misclassify themselves and the health effects of the two product types do not significantly differ [30]. Our own analysis confirmed no substantial risk difference between the two product types.

We included the leading causes of mortality identified by the Centers for Disease Control and Prevention (CDC) [31], although some are not generally associated with tobacco use (e.g., accidents), as well as other selected major mortality causes attributed to tobacco use (Table 1).

**Table 1:** Underlying cause of death and ICD-10 codes used for analysis

Cause of death		ICD 10 codes
Leading causes of death as assigned by CDC^a,b^	Diseases of the heart	I00-I09, I11, I13, I20-I151
	Heart failure	I50
	Ischemic heart disease	I20-I25
	Malignant neoplasm	C00-C97
	Chronic lower respiratory disease	J40-J47
	Cerebrovascular diseases	I60-I69
	Accidents	V01-X59, Y85-Y86
	Alzheimer’s disease	G30
	Diabetes mellitus	E10-E14
	Influenza and pneumonia	J09-J18
	Nephritis/nephrosis	N00-N07, N17-N19, N25-N27
Other selected causes of death	Malignant neoplasms:	
	All digestive organs	C00-C16, C18-C22, C25
	Esophagus	C15
	Pancreas	C25
	Colon, rectum, and anus	C18-C21
	Oral cavity, lip, and pharynx	C00-C14
	Trachea, bronchus, and lung	C33-C34
	Genitourinary system	C61, C64-C65, C67
	Diseases of the respiratory system	J00-J98
	Diseases of the digestive system	K00-K95
	Diseases of the genitourinary system	N00-N99

^a^National Vital Statistics Report (NVSR), Volume 64, Number 10. Table C. US Department Of Health and Human Services, Centers for Disease Control and Prevention, National Center for Health Statistics, National Vital Statistics System. Note: heart failure and ischemic heart disease are not included among the ten leading causes of death. We include them here for completeness

^b^Intentional self-harm, listed as a leading cause of death, was not identified in either data set and was therefore excluded from analysis

To protect respondent confidentiality, NCHS suppresses the reporting of HR estimates from the restricted NHIS data where the number of deaths is less than five. In some cases where the number of attributable mortalities for some endpoints did not exceed five within some tobacco use populations, our model using restricted access data did not compute HR estimates for that endpoint. While we do provide HR estimates for public data with counts less than five, we caution against the interpretation of these results since the low counts generally lead to wide confidence intervals.

## Results

### Sample demographics

Population demographics for the NHIS and NLMS survey data are shown in Additional file 3. Overall, there was reasonable agreement between the two surveys. The average age of enrollment ranged from 33.7–54.2-years old with little substantial difference between the NHIS and NLMS data; however, the average follow-up period for the NHIS survey (~ 150 months) was longer than the NLMS survey (~ 59 months). Current smokers, including current, former, or never SLT users, tended to have earlier deaths compared to former or never smokers. The age of death for never smokers who were current SLT users at enrollment was slightly lower than never-tobacco users, but they appeared to live longer by about 10 years than current smokers who never used SLT. SLT consumers, in general, tended to be predominantly white males with less education and lower family income compared to never-tobacco users.

### Impact of cigarette smoking and/or SLT use on major causes of death

Additional file 4 presents mortality risk estimates grouped according to common patterns of current cigarette and SLT use as measured in the surveys (exclusive cigarette smoker: current cigarette smoker/never SLT user; exclusive SLT user: never cigarette smoker/current SLT user; dual user: current cigarette smoker/current SLT user) compared to never-tobacco use.

**Fig. 1:**
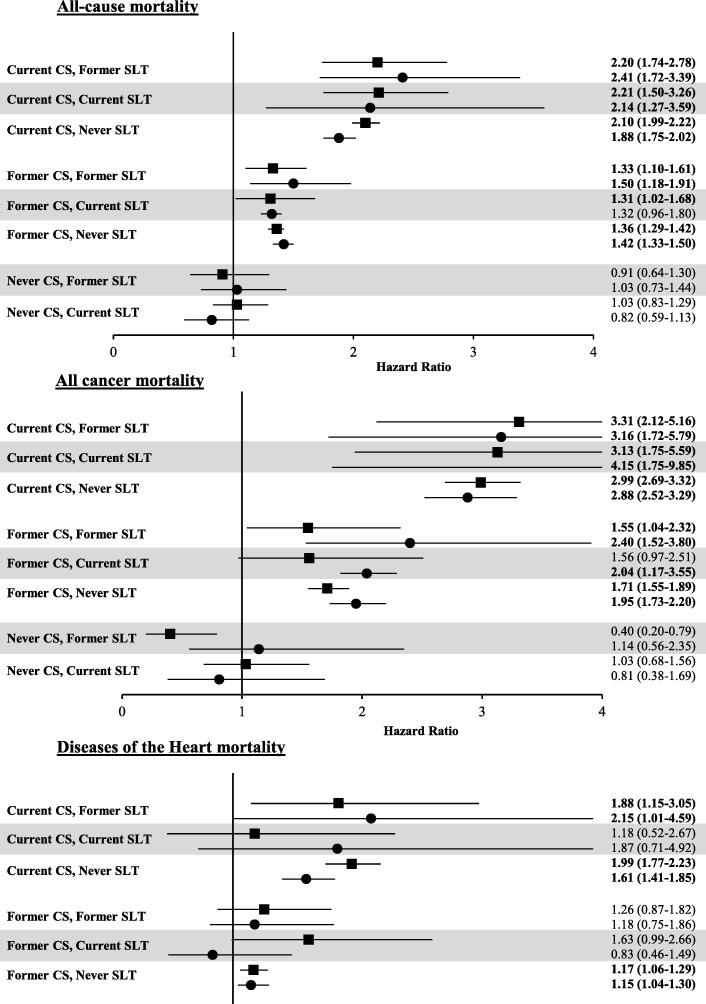
Impact of SLT use on mortality risks from all-causes, malignant neoplasms, and diseases of the heart among current, former, and never cigarette smokers (CS)

Data from both surveys indicated that exclusive smokers had significant excess risks for mortality from all causes, including diseases of the heart, cerebrovascular disease, malignant neoplasms, chronic lower respiratory diseases, influenza and pneumonia, and diseases of the digestive system (Additional file 4). In contrast, there was no evidence of excess mortality risk among exclusive SLT users (Additional file 4). However, it should be noted that the numbers of deaths used to derive the estimates were often very low and the confidence intervals were relatively wide. Overall, dual users had mortality risks consistent with exclusive cigarette smokers (excess mortality risk for all-cause mortality and malignant neoplasms). Additionally, mortality due to diseases of the heart among dual users was not statistically significantly elevated compared to never-tobacco use, which is in contrast to exclusive smokers. For endpoints where the data were particularly robust, such as all-cause mortality, diseases of the heart, and malignant neoplasms, the mortality risk estimates for exclusive SLT users generally clustered around 1.0 (Fig. 1).

### Impact of SLT use on mortality risk due to specific malignant neoplasms

Table 2 presents mortality risk estimates derived from NLMS datasets for several specific malignant neoplasms often attributed to SLT use and/or cigarette smoking. The limited number of attributable mortalities in the NHIS data prevented calculation of HR estimates with our model (see earlier comments in the “Methods” section).

**Table 2:** Estimated mortality risk^a^ from specific malignant neoplasms according to cigarette or SLT use (NLMS data)

Cause of death	HR (95% CI)^b^ [number of deaths]		
	Exclusive smokers (current smoker/never SLT: 38,076 observations)	Exclusive SLT user (current SLT/never smoker: 1863 observations)	Dual user (current smoker/current SLT: 657 observations)
Trachea, bronchus, and lung	*11.52 (8.74–15.19)*^c^ [247]	2.982 (0.91–9.76) [3]	*11.46 (3.31–39.6)* [3]
Oral cavity, lip, and pharynx	*6.33 (1.46–27.38)* [9]	NA^d^ [0]	NA [0]
All digestive organs	*1.75 (1.31–2.34)* [89]	1.01 (0.32–3.20) [3]	NA [0]
Esophagus	*2.31 (1.10–4.85)* [16]	2.44 (0.31–19.1) [1]	NA [0]
Pancreas	1.48 (0.86–2.56) [24]	1.36 (0.19–9.98) [1]	NA [0]]
Colon, rectum, and anus	1.47 (0.91–2.35) [30]	NA [0]]	NA [0]]
Genitourinary system	*2.10 (1.34–3.31)* [37]	0.51 (0.07–3.78) [1]	2.77 (0.37–20.7) [1]

^a^Analysis included all respondents from NLMS public data version 5 and NHIS restricted access data

^b^The reference group comprised individuals who never used tobacco (according to survey defined parameters)

^c^Italicized risk estimates denote statistical significance (CI estimates do not include 1.0)

^d^*NA* not applicable

*CI* confidence interval, *HR* hazard ratio, *NHIS* National Health Interview Survey; *NLMS* National Longitudinal Mortality Study, *SLT* smokeless tobacco

Consistent with previous literature [32, 33], exclusive cigarette smokers had an increased mortality risk from cancers of the respiratory tract, oral cavity, digestive organs, esophagus, and genitourinary system (Table 2). Mortality from these cancers was rare in exclusive SLT users in both data sets. Death from respiratory tract diseases in smokers accounts for about half of smoking-related mortalities each year [34]. Consistent with this, risk of dying from cancers of the trachea, bronchus, or lung was elevated among exclusive smokers and dual users (Table 2). In contrast, exclusive SLT users demonstrated no increased mortality risk from respiratory tract cancers.

Mortality risk from oral cancer among SLT users was not measurable in either dataset because there were too few deaths in this group to derive a risk estimate. Within the restricted NHIS data, there were less than five deaths due to cancers of the oral cavity, lip, and pharynx among SLT users and none among SLT users in the NLMS data. We note that almost all instances of death due to oral cancer occurred among current or former cigarette smokers.

### Impact of SLT use on mortality risk from certain circulatory diseases

There are reports in the published literature of associations between SLT use and excess risk for certain circulatory diseases, particularly ischemic heart disease (IHD) [3, 13, 35]. As shown in Table 3, we did not detect significantly elevated mortality risks among SLT users for the broad category of diseases of the heart in either the NHIS or NLMS data sets nor did we detect significantly elevated risks for IHD (ICD-10 codes I20-I25) mortality among SLT users. This was surprising as Timberlake et al. reported excess IHD mortality among exclusive SLT users based on analysis of the NLMS data set [13]. Possible reasons for this discrepancy are discussed below. However, we did find excess mortality risk for heart failure (ICD-10 code I50) among current SLT users in the restricted access NHIS data set (note this mortality outcome is not available in the NHIS public use file). We did not replicate this finding in the NLMS data set, although there were only two heart failure deaths among current SLT users in the NLMS data set, so we do not regard this result as robust.

**Table 3:** Risk estimates calculated for heart failure and ischemic heart disease among current exclusive SLT users

Cause of Death (ICD-10 Codes)	Hazard ratio (95% CI)^a^ [number of deaths]	
	NLMS (1863 observations)	NHIS (1561 observations)
Diseases of the heart (I00-09, I11, I13, I20-51)	1.07 (0.65–1.75) [22]	1.20 (0.91–1.58) [114]
Heart failure (I50)	1.13 (0.28–4.62) [2]	*2.75 (1.55–4.89)*^b^ [21]
Ischemic heart disease (I20-25)	0.95 (0.49–1.83) [14]	1.06 (0.75–1.49) [79]

^a^The reference group comprised individuals who never used tobacco (according to survey defined parameters)

^b^Italicized risk estimates denote a statistically significant difference between the test group noted and a no-tobacco reference group

### Impact of SLT use on reduced health risk arising from smoking cessation

Additional file 5 shows the estimated all-cause mortality risk and mortality risk for all cancers and diseases of the heart for exclusive smokers, former smokers, and never smokers, combined with various levels of SLT use (current, former, and never). Exclusive smokers had the greatest risk for any of the endpoints measured, which is consistent with previous data [36]. Of note, smoking cessation greatly reduced these mortality risks. Former smokers who were current SLT users at the time of interview did not have increased mortality risk compared to current smokers who are never SLT users in the NLMS data set.

## Discussion

Public health agencies, including the United States Food and Drug Administration, acknowledge a continuum of risk among tobacco products, with combustible tobacco products (e.g., cigarettes) being the most harmful along this continuum and noncombustible tobacco products (e.g., SLT) being less harmful [6, 8, 37, 38]. Cigarette smoking is the leading preventable cause of death in the USA, primarily due to lung cancer, respiratory disease, and cardiovascular disease [2]. Studies with SLT users demonstrate that SLT products available in the USA may reduce the harm from cigarette smoking by providing a substantially lower risk alternative to those smokers who are unable or unwilling to cease using tobacco products [39–42]. However, others question the utility of SLT products in a harm reduction strategy, since these products are reported to be associated with cancers of the upper aerodigestive tract, particularly the oral cavity, esophagus, and pancreas, and cardiovascular diseases [43].

Much of the scientific concern regarding the health risks associated with SLT use relies on epidemiological studies performed several decades ago, and risks associated with tobacco product use may change over time [11]. Many SLT products available worldwide are associated with serious and often fatal diseases [1]. Globally, a wide variety of SLT products are used, many of which can be used on their own, mixed with other products (such as slaked lime [khaini]), or used as ingredients in other products (such as betel quid). In these situations, interpretation of results is complicated since study participants might be exposed to products with variable amounts of carcinogens. For example, the levels of the tobacco-specific nitrosamines are 100-fold higher in the SLT product, “Toombak”, which is available in Sudan, compared to US products [44].

In the USA, the dominant form of tobacco use was chewing tobacco until the 1920s after which, smoking of machine-made cigarettes became prevalent [45]. In the 1970s, a resurgence in SLT products occurred mainly in the form of chewing tobacco (predominantly loose leaf). MST products, although in existence since 1822, gained popularity and surpassed sales of chewing tobacco after 1980 [46] and today, represent approximately 85% of the SLT market in the USA. In a previous analysis, Henley et al. utilized data from participants in the Cancer Prevention Study (CPS)-I and CPS-II studies; participants in the CPS-I study were enrolled in 1959 with follow-up 12 years later in 1971 whereas participants in the CPS-II study were enrolled in 1982 with follow-up 18 years later in 2000 [3]. The goal of the current analysis was to supplement the collection of SLT epidemiological evidence with an updated analysis based on current health risk survey data collected through 2011. The NMLS and the NHIS are nationally representative surveys of the civilian non-institutionalized US population representing the years 1986 through 2009. These surveys are linked to NDI data, and provide vital longitudinal follow-up through December 31, 2011. Based on the NMLS and NHIS datasets, SLT users, in general, did not display a significantly increased risk for all-cause mortality, all-cancer mortality, or diseases of the heart compared to never-tobacco users. The all-cause mortality risks derived from our analysis of respondents in the NHIS and NLMS data sets are consistent with those found in the published scientific literature. The all-cause mortality HR (95% CI) for SLT users is estimated as 1.0 (0.8–1.3) for males and 1.3 (0.9–1.7) for females [3].

Additionally, SLT use had no discernable adverse effect on any of the nine leading causes of death and did not increase mortality risk for any of the major neoplasms often associated with SLT use. We noted clear indications of increased mortality risks in cigarette smokers due to all-causes, all-cancers, and diseases of the heart. The magnitudes of the excess mortality risks among cigarette smokers for the endpoints measured were very consistent between the two independently linked mortality datasets and generally consistent with those documented in the published literature [11, 34]. Previous research has shown that the health risks of dual users of SLT and cigarettes are similar to those of exclusive cigarette smokers [47]. Although there were limited numbers of deaths in the data sets used for the current analysis, we noted similar findings. We also noted that smoking cessation leads to a noticeable reduction in, but not necessarily elimination of, excess mortality risk. Here again, based on a limited sample size, our analysis found minimal or no adverse effect of SLT on this trend.

Some public health authorities have concluded that SLT use is causally associated with oral, esophageal, and pancreatic cancers [2]. Both the NMLS and NHIS datasets recorded an insufficient number of oral cancer mortality events to provide derivation of a mortality risk estimate for current SLT users. Notably, the leading cause of cancer-related mortality among smokers (about 12-fold increase over background in the two datasets analyzed) was virtually non-existent for SLT users in the NLMS and NHIS datasets.

Our results are generally concordant with those of Timberlake et al. who analyzed NMLS data and detected no excess risk of all-cause mortality, or mortality from all cancers, cerebrovascular disease, or cancers of the digestive organs or pancreas [13]. However, these authors did report an excess mortality risk for IHD (reported as coronary heart disease) (HR 1.24, 95% CI 1.05–1.46). We detected no excess diseases of the heart mortality risk due to SLT use in either dataset we examined (NLMS HR 0.82 (0.51–1.13 and NHIS HR 1.03 (0.83–1.29)) nor did we detect a significantly excess risk of mortality due to IHD in either NLMS or NHIS. However, our point estimate for IHD among current SLT users in the NLMS data set is 1.25, which is almost identical to the estimate of 1.24 reported by Timberlake et al. Our result was based on 14 IHD deaths among 1863 current SLT users while Timberlake’s was based on 86 deaths among 4919 current SLT users. Timberlake et al. analyzed the restricted access NLMS file, which included data from 1986 to 2011, while we analyzed the more limited public use NLMS file, which included data from 1993 to 2005. The difference in survey years included in the data analyzed by Timberlake et al. compared to the current study accounts for the difference in sample size and the larger confidence interval surrounding our estimate.

Our analysis is not without limitations similar to many long-term epidemiological studies. Possible misclassification of tobacco use practices, inconsistencies in tobacco use category assignments, and self-reporting of tobacco use are common problems in epidemiology studies of tobacco use. Further, reliance on baseline assessment to assign respondent tobacco use status can result in misclassification and does not fully capture all changes in tobacco use. Additionally, the follow-up period may not be long enough to completely cover the “latency period” for development of tobacco-related cancers, e.g., a 20-year latency period is often reported for lung cancer [48]. Nonetheless, our analyses identified excess health risks associated with cigarette smoking which were in close approximation with many other published studies [34]. We believe the close agreement of our results for current and former cigarette smokers with the recently published data, the concordance of two independent datasets, and the large number of observations in each data set all lend credibility to the results we observed for other tobacco use behaviors, including exclusive SLT use and dual use.

The significant health risks of cigarette smoking are well established, with over 400,000 attributable deaths estimated to occur in the USA each year [34]. Some in the public health arena have proposed a broader consideration of SLT products as a safer alternative to cigarette smoking [49]. In its 2007 report, the Royal College of Physicians Tobacco Advisory Group called for an evidence-based regulatory approach to SLT and harm reduction [38]. The evidence from our analyses supports this approach. Switching to lower risk products could provide a benefit to those adult smokers who are unable or unwilling to quit cigarettes. However, harm reduction can only be achieved if adult cigarette smokers who wish to continue using tobacco completely switch to SLT. For some, this conversion is inhibited by the belief that SLT use is as harmful as cigarette smoking; only 9% of adults in the USA consider some SLT to be safer than smoking [50, 51].

## Conclusions

Emerging epidemiological data are helpful in evaluating the health risks of current SLT product use compared to cigarette smoking. Our analysis of two current US datasets measuring the effects of contemporary tobacco products demonstrates a clear mortality risk differential between use of current SLT products and cigarette smoking. Cigarette smokers in the surveys had an increased overall mortality risk and an increased risk for several specific causes of death (e.g., lung cancer), while SLT users consistently had comparatively lower mortality risks.

## Additional files

Additional file 1: Accounting of records in the NLMS Data. (PDF 92 kb)

Additional file 2: Accounting of records in the NHIS Data. (PDF 91 kb)

Additional file 3: Sample and demographic characteristics of tobacco use groups. (PDF 195 kb)

Additional file 4: Estimated mortality risk from all-causes and specific diseases according to cigarette or SLT use. (PDF 39 kb)

Additional file 5: Estimated mortality risk for current and former smokers. (PDF 34 kb)

## Abbreviations

CDC: Centers for Disease Control and Prevention
CPS: Cancer Prevention Studies
CPS-TUS: Current Population Survey Tobacco Use Supplements
HR: Hazard ratio
MST: Moist smokeless tobacco
NCHS: National Center for Health Statistics
NDI: National Death Index
NHIS: National Health Interview Survey
NLMS: National Longitudinal Mortality Study
PUMS: Public Use Microdata Sample
SLT: Smokeless tobacco
TU: Tobacco use
US: United States
