# Supplementary material for: Smokeless tobacco mortality risks: an analysis of two contemporary nationally representative longitudinal mortality studies
Source: Harm Reduct J. 2019 Apr 11;16:27. doi: 10.1186/s12954-019-0294-6 (PMC6458834; doi:10.1186/s12954-019-0294-6)
Supplement: Supplementary file 1 — Accounting of records in the NLMS Data. (PDF 92 kb) [file 12954_2019_294_MOESM1_ESM.pdf]

Additional File 1: Accounting of Records in the NLMS Data

	Total	Male	Female	Percent drop from pervious subset
Total records	493,282	230,967	262,315	-
Limited to age 18+	465,271	216,589	248,682	5.7
Limited to those who answered tobacco use questions	408,438	189,645	218,793	12.2
Limited to those who never smoked pipes or cigars	375,690	160,620	215,070	8.0
Limited to records with complete tobacco use status information	375,552	160,557	214,995	0.0
Limited to records with known weights and mortality follow-up	375,197	160,399	214,798	0.1
Limited to records with known education and income variables	370,640	158,271	212,369	1.2
Limited to records with known health status at baseline	210,090	87,166	122,924	43.3
