# Supplementary material for: Smokeless tobacco mortality risks: an analysis of two contemporary nationally representative longitudinal mortality studies
Source: Harm Reduct J. 2019 Apr 11;16:27. doi: 10.1186/s12954-019-0294-6 (PMC6458834; doi:10.1186/s12954-019-0294-6)
Supplement: Supplementary file 2 — Accounting of records in the NHIS Data. (PDF 91 kb) [file 12954_2019_294_MOESM2_ESM.pdf]

Additional File 2: Accounting of Records in the NHIS Data

	Total	Male	Female	Percent drop from pervious subset
Total records	669,355	321,001	348,354	-
Limited to age 18+	479,872	224,352	255,520	26.6
Limited to those who answered tobacco use questions	195,108	83,362	111,746	56.3
Limited to those who never smoked pipes or cigars	171,778	63,511	108,267	3.1
Limited to records with complete tobacco use status information	169,295	62,570	106,725	1.4
Limited to records linkable to National Death Index	163,672	60,469	103,203	3.3
Limited to records with completed variables for Age, Sex, BMI, Education, Family Income, Health Status at Baseline, stratification and weighting variables, and death data	154,391	57,762	96,629	6.4
