# Supplementary material for: Smokeless tobacco mortality risks: an analysis of two contemporary nationally representative longitudinal mortality studies
Source: Harm Reduct J. 2019 Apr 11;16:27. doi: 10.1186/s12954-019-0294-6 (PMC6458834; doi:10.1186/s12954-019-0294-6)
Supplement: Supplementary file 3 — Sample and demographic characteristics of tobacco use groups. (PDF 195 kb) [file 12954_2019_294_MOESM3_ESM.pdf]

Additional file 3. Sample and demographic characteristics of tobacco use groups

A. NHIS[a]	Current Smokers			Former Smokers			Never Smokers		
Group number	1	2	3	4	5	6	7	8	9
	Current SLT users	Former SLT users	Never SLT users	Current SLT users	Former SLT users	Never SLT users	Current SLT users	Former SLT users	Never SLT users
Sample size	699	1,599	36,114	745	1,184	28,564	1,562	1,083	82,841
No. of deaths	99	255	7,450	205	310	7,363	338	138	13,285
Avg. follow-up quarters	51.9	49.1	50.4	46.9	46.1	46.8	49.0	49.6	48.7
Avg. age at interview	34.1	35.7	41.6	46.5	46.6	51.9	41.3	37.3	44.2
% Male	92.7	87.9	37.5	90.6	88.5	39.0	78.2	85.1	32.8
% White race/ethnicity	89.3	87.9	80.1	90.1	87.3	86.4	79.4	86.6	79.2
% Some college or more	29.8	32.3	34.5	28.3	39.2	46.5	35.9	55.6	50.4
% Family income ≥ $20,000	59.4	60.4	57.5	61.1	65.1	68.9	54.3	66.9	64.9
Avg. body mass index	25.8	25.7	25.2	27.1	27.1	26.4	26.7	26.7	25.9
% Good health or better	85.4	84.3	84.5	80.0	82.8	84.5	81.0	91.0	89.4
Avg. cigarettes per day	15.6	19.0	16.2	23.5	22.9	20.2	-	-	-
B. NLMS[b]	Current Smokers			Former Smokers			Never Smokers		
Group number	1	2	3	4	5	6	7	8	9
	Current SLT users	Former SLT users	Never SLT users	Current SLT users	Former SLT users	Never SLT users	Current SLT users	Former SLT users	Never SLT users
Sample size	657	1,464	38,076	972	1,379	39,401	1,863	1,821	124,457
No. of deaths	22	49	1,505	59	91	2,703	48	48	4,055
Avg. follow-up months	59.7	59.8	59.6	58.8	58.6	58.6	60.2	60.1	59.8
Avg. age at interview	36.2	36.0	42.5	47.3	47.7	53.3	39.6	39.7	44.6
% Male	92.8	80.0	43.6	94.0	85.7	43.2	88.4	83.5	37.4
% White race/ethnicity	91.3	88.7	85.1	94.8	90.9	89.5	87.9	89.2	82.6
% Some college or more	33.9	36.7	37.8	32.8	46.0	49.7	41.1	60.6	54.4
% Family income ≥ $20,000	67.1	62.3	63.2	73.4	70.8	72.2	70.5	79.6	72.3
% Good health or better	87.1	86.0	83.6	80.6	81.9	81.9	84.8	90.5	87.9

[a] NHIS 1987, 1991, 1992, 1998, 2000 and 2005 surveys; restricted-access NHIS linked mortality files with follow-up through 12/31/2011 (public use file, note ever cigar and pipe smokers are excluded).
[b] Source: Public-Use NLMS Version 5 Tobacco Use File.
Note: NHIS: National Health Interview Survey; NLMS National Longitudinal Mortality Study, SLT = Smokeless tobacco
