# Supplementary material for: Smokeless tobacco mortality risks: an analysis of two contemporary nationally representative longitudinal mortality studies
Source: Harm Reduct J. 2019 Apr 11;16:27. doi: 10.1186/s12954-019-0294-6 (PMC6458834; doi:10.1186/s12954-019-0294-6)
Supplement: Supplementary file 4 — Estimated mortality risk from all-causes and specific diseases according to cigarette or SLT use. (PDF 39 kb) [file 12954_2019_294_MOESM4_ESM.pdf]

Additional file 4. Estimated mortality risk[a] from all-causes and specific diseases according to cigarette or SLT use.

Cause of Death	HR (95% CI)[b] [Number of Deaths]					
	Exclusive smokers (current smoker/never SLT)		Exclusive SLT user (current SLT/never smoker)		Dual user (current smoker/current SLT)	
	NHIS (36,114 observations)[c]	NLMS (38,076 observations)	NHIS (1,562 observations)	NLMS (1,863 observations)	NHIS (699 observations)	NLMS (657 observations)
All-cause mortality	**2.10 (1.99-2.22)**[d] [3,758]	**1.88 (1.75-2.02)** [1,505]	1.03 (0.83-1.29) [193]	0.82 (0.59-1.13) [48]	**2.21 (1.50-3.26)** [50]	**2.14 (1.27-3.59)** [22]
Diseases of the heart	**1.99 (1.77-2.23)** [775]	**1.61 (1.41-1.85)** [378]	0.86 (0.59-1.26) [52]	1.07 (0.65-1.76) [22]	1.18 (0.52-2.67) [7]	1.86 (0.71-4.92) [5]
Chronic lower respiratory diseases	**10.97 (8.43-14.28)** [294]	**6. 26 (4.54-8.63)** **[111]**	0.43 (0.10-1.78) [2]	NA [0]	7.06 (2.55-19.54) [3]	NA [0]
Malignant neoplasms	**2.99 (2.69-3.32)** [1,164]	**2.88 (2.52-3.29)** [520]	1.03 (0.68-1.56) [42]	0.81 (0.38-1.70) [8]	**3.13 (1.75-5.59)** [18]	**4.15 (1.75-9.85)** [7]
Cerebrovascular diseases	**1.61 (1.31-1.99)** [187]	**1.71 (1.27-2.30)** [781]	0.65 (0.31-1.34) [10]	0.60 (0.19-1.92) [3]	3.97 (0.93-16.94) [3]	3.97 (0.93-16.94) [3]
Accidents (unintentional injuries)	**1.67 (1.26-2.23)** [139]	1.20 (0.81-1.80) [52]	1.07 (0.44-2.59) [9]	0.38 (0.07-1.95) [2]	1.57 (0.37-6.63) [2]	1.56 (0.37-6.63) [2]]
Alzheimer's disease	0.99 (0.50-2.00) [16]	1.08 (0.43-2.74) [8]	1.49 (0.38-5.82) [3]	1.95 (0.28-13.61) [1]	NA [0]	NA [0]
Diabetes mellitus	1.00 (0.69-1.45) [72]	1.31 (0.85-2.04) [39]	1.04 (0.27-4.07) [6]	1.47 (0.36-5.96) [2]	3.43 (0.71-16.69) [2]	NA [0]
Influenza and pneumonia	**2.25 (1.58-3.19)** [89]	1.04 (0.64-1.69) [28]	1.16 (0.53-2.55) [7]	NA [0]	1.00 (0.13-7.465) [1]	5.63 (0.74-42.68) [1]
Nephritis, nephrotic syndrome, and nephrosis	0.88 (0.54-1.43) [35]	1.36 (0.66-2.80) [16]	0.81 (0.27-2.42) [4]	NA [0]	7.50 (0.98-57.6) [1]	NA [0]

[a] Analysis included all respondents from NLMS public data version 5 and NHIS public access 10 year follow-up data.
[b] The reference group comprised individuals who never used tobacco (according to survey defined parameters).
[c] Total observation data shown is for all-cause mortality. In some cases, observations for other diseases may be slightly less.
[d] Bolded risk estimates denote statistical significance (CI estimates do not include 1.0).
[e] NA = not applicable
CI = confidence interval, HR = hazard ratio, NHIS: National Health Interview Survey; NLMS National Longitudinal Mortality Study, SLT = smokeless tobacco.
