# Supplementary material for: Smokeless tobacco mortality risks: an analysis of two contemporary nationally representative longitudinal mortality studies
Source: Harm Reduct J. 2019 Apr 11;16:27. doi: 10.1186/s12954-019-0294-6 (PMC6458834; doi:10.1186/s12954-019-0294-6)
Supplement: Supplementary file 5 — Estimated mortality risk for current and former smokers. (PDF 34 kb) [file 12954_2019_294_MOESM5_ESM.pdf]

Additional file 5. Estimated mortality risk[a] for current and former smokers.

Cause of Death	HR (95% CI)[b] [Number of Deaths]					
	Current exclusive smokers (current smoker/never SLT)		Formers smokers			
			no current SLT use		current SLT use	
	NHIS (36,114 observations)[c]	NLMS (38,076 observations)	NHIS (16,043 observations)	NLMS (8,580 observations)	NHIS (16,043observations)	NLMS (8580 observations)
All-cause mortality	**2.10 (1.99-2.22)[d]** [3,758]	**1.88 (1.75-2.02)** [1,505]	**1.36 (1.29-1.42)** [4,348]	**1.416 (1.334-1.50)** [2,703]	**1.31 (1.02-1.68)** [127]	1.317 (0.96-1.802) [59]
Diseases of the heart	**1.99 (1.77-2.23)** [775]	**1.61 (1.41-1.85)** [378]	**1.17 (1.06-1.29)** [1,006]	**1.16 (1.04-1.30)** [794]	1.63(0.99-2.66) [39]	0.83 (0.46-1.49) [14]
Chronic lower respiratory diseases	**10.97 (8.43-14.28)** [294]	**6. 26 (4.54-8.63)** **[111]**	6.55 (5.01-8.56) [347]	**4.98 (3.82-6.49)** [239]	1.56 (0.97-2.51) [30]	**2.04 (1.73-3.55)** [19]
Malignant neoplasms	**2.99 (2.69-3.32)** [1,164]	**2.88 (2.52-3.29)** [520]	1.71 (1.56-1.89) [1,121]	**1.95 (1.73-2.20)** [758]	**4.08 (2.02-8.24)** [11]	**2.02 (0.59-6.92)** [3]
Cerebrovascular diseases	**1.61 (1.31-1.99)** [187]	**1.71 (1.27-2.30)** [781]	1.00 (0.82-1.21) [235]	1.15 (0.92-1.45) [177]	2.00 (0.83-4.83) [8]	**3.46 (1.27-9.41)** [6]

[a] Analysis included all respondents from NLMS public data version 5 and NHIS public access 10-year follow-up data.
[b] The reference group comprised individuals who never used tobacco (according to survey defined parameters).
[c] Total observation data shown is for all-cause mortality. In some cases, observations for other diseases may be slightly less.
[d] Bolded risk estimates denote statistical significance (CI estimates do not include 1.0).
CI = confidence interval, HR = hazard ratio, NHIS: National Health Interview Survey; NLMS National Longitudinal Mortality Study, SLT = smokeless tobacco
